# Mesenteric extraovarian Sertoli-Leydig cell tumor without *DICER1* hotspot mutation: a case report

**DOI:** 10.1186/s13000-019-0805-5

**Published:** 2019-04-01

**Authors:** Mitsumasa Osakabe, Chie Sato, Masamichi Suzuki, Ryo Sugimoto, Yasuko Fujita, Noriyuki Uesugi, Kazuyuki Ishida, Hiroaki Itamochi, Tsukasa Baba, Tamotsu Sugai

**Affiliations:** 1 Department of Molecular Diagnostic Pathology, School of Medicine, Iwate Medical University, 19-1, Morioka, 020-8505 Japan; 2 Department of Obstetrics and Gynecology, School of Medicine, Iwate Medical University, 19-1, Morioka, 020-8505 Japan

**Keywords:** Mesentery, Sertoli-Leydig cell tumor, Heterotopic tumor, *DICER1*

## Abstract

**Background:**

Ovarian Sertoli-Leydig cell tumors (SLCTs) with androgenic manifestations harbor *DICER1* mutations in 30–60% of cases. Ovarian SLCTs without *DICER1* hotspot mutations have been reported to exhibit elderly onset and no androgenic manifestations. We present the first case of a primary mesenteric SLCT without *DICER1* hotspot mutation.

**Case presentation:**

An 84-year-old woman presented with a 75-mm mesenteric solid tumor. She presented no androgenic or estrogenic manifestations. She underwent ileocecal resection. Histologically, her mesenteric tumor showed histopathological features that resembled moderately differentiated SLCT. Moreover, *DICER1* hotspot mutation was not detected.

**Conclusions:**

We described the first case of heterotopic primary mesenteric SLCT without *DICER1* hotspot mutation.

## Background

Sertoli-Leydig cell tumors (SLCTs) are rare ovarian tumors that belong to the group of sex-cord stromal tumors, comprising less than 0.5% of ovarian neoplasms, and mainly occur in young adult women in their 20s [1]. The occurrence of primary sex-cord stromal tumors at heterotopic extraovarian sites is exceptionally rare [2, 3]. Many heterotopic extraovarian sex-cord stromal tumors occur in the broad ligament and retroperitoneum, and these histological types are granulosa cell tumors [2, 3]. To the best of our knowledge, only one case of primary mesenteric SLCT has been reported [3].

Ovarian SLCTs have been extensively studied owing to their androgenic manifestations. Although ovarian SLCTs show masculinization in approximately 40–60% of cases, the remaining cases are nonfunctional or estrogen producing [1, 4, 5]. However, hormone production by primary mesenteric SLCT has not been evaluated [3].

Recently, germline mutations in *DICER1*, which encodes a ribonuclease III endonuclease that contributes to the biosynthesis of microRNAs, have been identified in families affected by pleuropulmonary blastomas, multinodular goiter, and ovarian SLCTs [6–8]. Somatic mutations in *DICER1* are also found in 30–60% of sporadic cases of ovarian SLCTs [9–12]. *DICER1* hotspot-mutated ovarian SLCTs are characterized by androgenic effects, earlier age of onset, and frequent relapse compared with ovarian SLCTs without *DICER1* hotspot mutation [12, 13].

Here, we describe the first case of a primary mesenteric extraovarian SLCT without *DICER1* hotspot mutation.

## Case presentation

An 84-year-old woman underwent total hysterectomy, bilateral salpingo-oophorectomy, and omentectomy 18 years ago because of right ovarian fibrothecoma. After surgery, she was followed up regularly. During follow-up, she presented no androgenic or estrogenic manifestations. She was found to have a pelvic tumor that was suspected to be recurrence of fibrothecoma. Her pelvic tumor was located in the mesentery of the distal ileum. She underwent ileocecal resection to remove the mesenteric tumor. She received no additional therapy. She had an uneventful postoperative course and no recurrence for 1 year after surgery.

### Pathological findings of the right ovarian tumor from 18 years ago

**Fig. 1:**
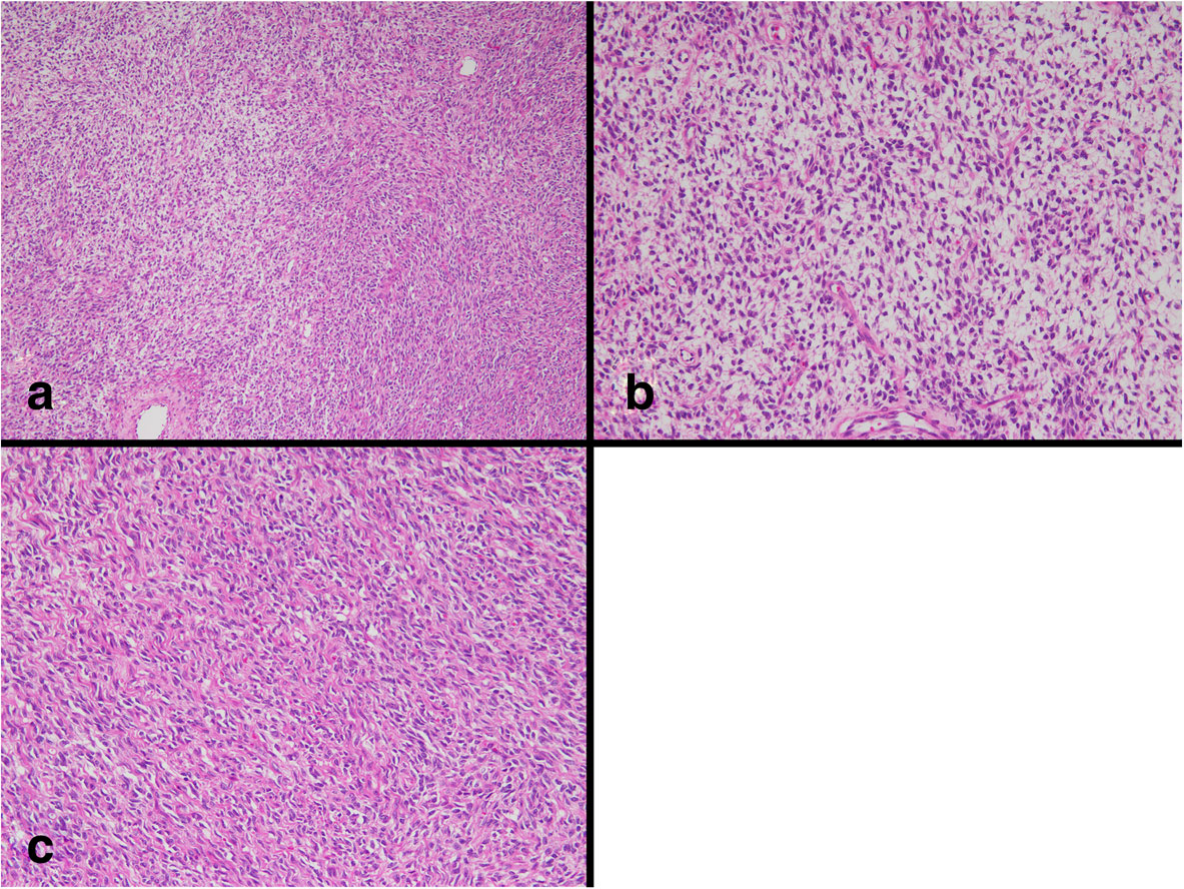
Histopathological findings of the right ovarian tumor harvested 18 years prior. **a** Low-power view. **b** Growth of theca cell-like cells. **c** Fibroma-like area

We re-evaluated the right ovarian tumor collected 18 years ago. The right ovarian tumor was a yellowish white solid tumor the size of an adult head. Fourteen tissue sections were prepared from the right ovarian tumor. Microscopically, theca cell-like cells and collagen-producing fibroblasts were observed in all tissue specimens (Fig. 1a–c). No SLCT component was observed in any of the tissue specimens. We diagnosed the patient with fibrothecoma as a result of re-evaluation.

### Pathological findings of the mesenteric tumor

**Fig. 2:**
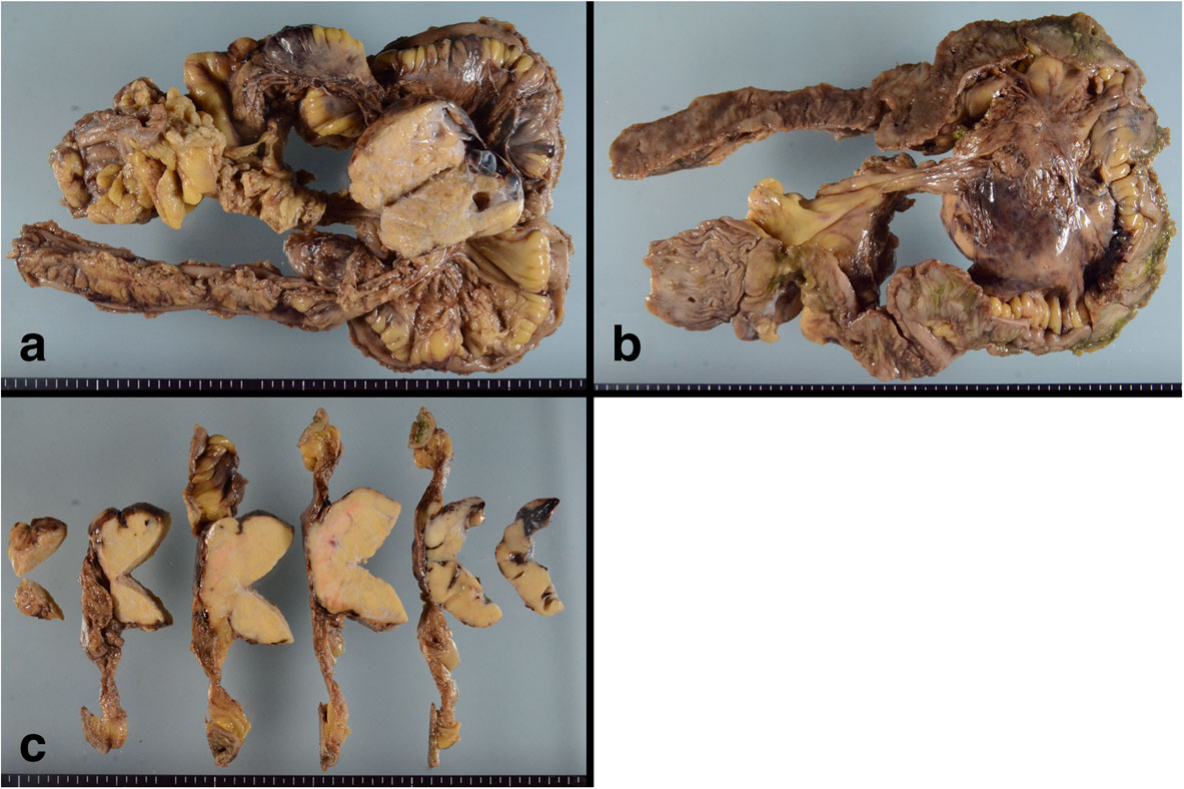
Macroscopic findings of the mesenteric tumor. **a**, **b** Nodular and well circumscribed tumor in the mesentery. **c** The cut surface of the mesenteric tumor

**Fig. 3:**
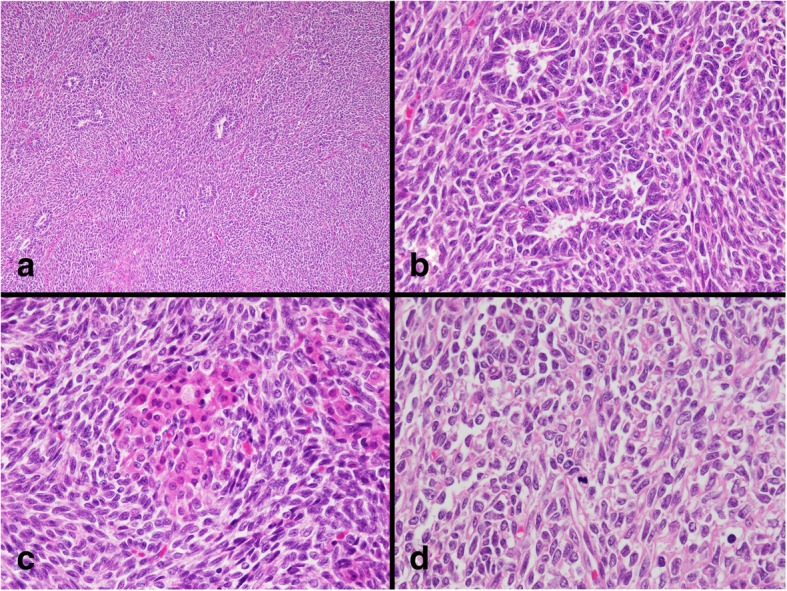
Histopathological findings of the mesenteric tumor. **a** Low-power view. **b** Tubules consisted of Sertoli cell-like tall columnar cells. **c** Nests of Leydig cell-like eosinophilic cuboidal cells. **d** A few mitotic cells were observed

Macroscopically, the mesenteric tumor was nodular and well circumscribed, measuring 75 × 65 × 50 mm (Fig. 2a, b). The cut surface was yellow (Fig. 2c). The tumor did not invade into the ileal wall. Microscopically, duct-like structures, which consisted of Sertoli cell-like tall columnar cells, were observed in the diffuse growth of scant cytoplasmic ovoid cells (Fig. 3a, b). Additionally, nests of Leydig cell-like cuboidal cells with eosinophilic cytoplasm were observed (Fig. 3c). The mitotic rate of the tumor was 2 per 10 high-power fields (Fig. 3d). No heterologous elements were observed.

**Fig. 4:**
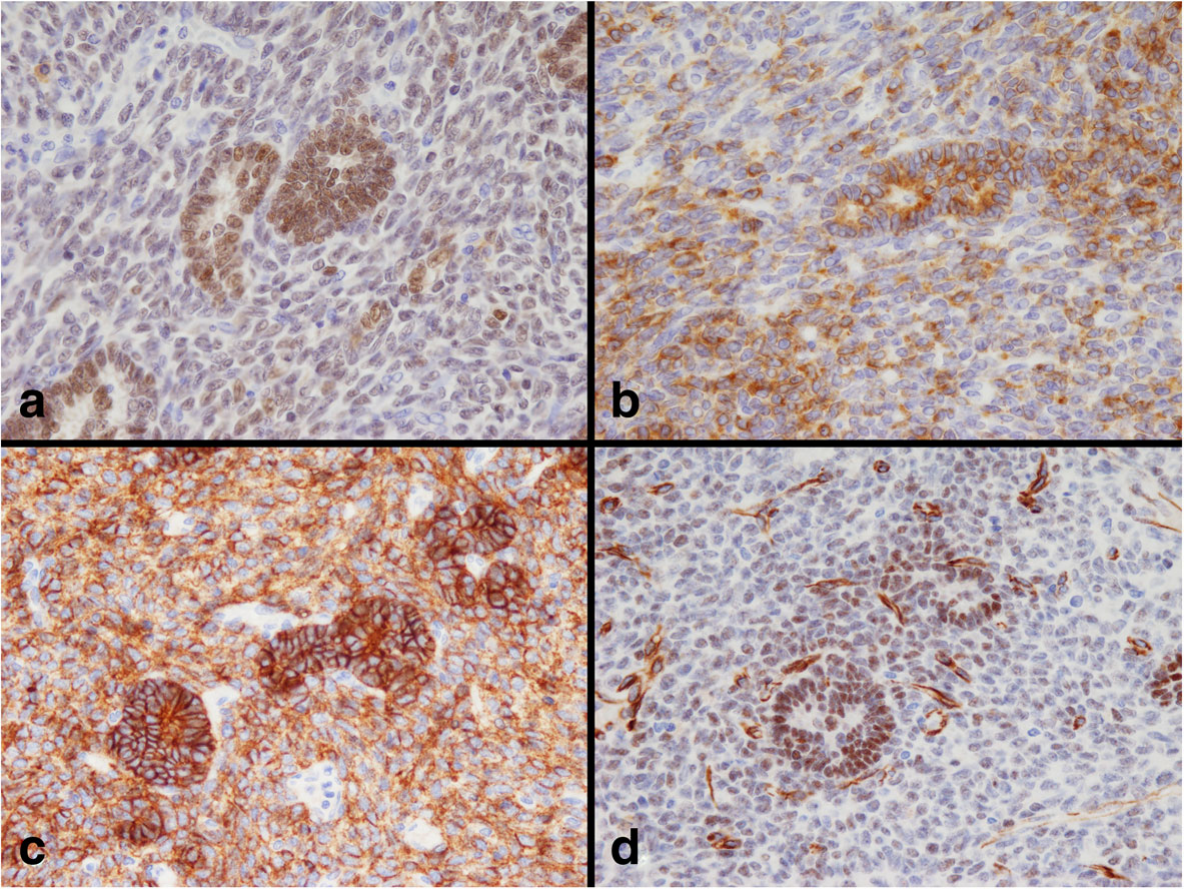
Immunohistochemical findings of the mesenteric tumor. **a** SF-1, (**b**) inhibin-α, (**c**) CD56, (**d**) WT-1

An automatic staining machine (DAKO Envision+ system; DakoCytomation, Glostrup, Denmark) was used for the immunohistochemical procedure. The antibodies used in this study are shown in Table 1. Positive immunohistochemical expression of steroidogenic factor-1 (SF-1; Fig. 4a), inhibin-α (Fig. 4b), cluster of differentiation 56 (CD 56; Fig. 4c), Wilms tumor 1 (WT-1; Fig. 4d), AE1/AE3, and vimentin was found in Sertoli cell-like tall columnar cells. Inhibin-α and vimentin were expressed in Leydig cell-like cuboidal cells. Positive expression of SF-1, inhibin-α, CD56, and vimentin was found in ovoid cells.

**Table 1:** List of primary antibodies

Antibody	Clone	Supplier	Dilution
Anti-SF-1	N1665	Thermo Fisher	1:20
Anti-inhibin-α	R1	DAKO	1:50
Anti-CD56	123C3	DAKO	No dilution
Anti-WT-1	6F-H2	DAKO	No dilution
Anti-AE1/AE3	AE1/AE3	DAKO	No dilution
Anti-vimentin	V9	DAKO	No dilution

Finally, we examined hotspot mutations in exons 24 and 25 encoding the *DICER1* ribonuclease IIIb domain using a direct sequencing method. Before DNA extraction, neoplastic cells accounted for at least 50% of the tissue cell population. DNA was extracted from formalin-fixed, paraffin-embedded tissues. No *DICER1* hotspot mutation was detected in this tumor tissue.

We therefore diagnosed the patient with primary mesenteric moderately differentiated SLCT without *DICER1* hotspot mutation.

## Discussion and conclusion

The occurrence of primary sex-cord stromal tumors at heterotopic extraovarian sites is exceptionally rare [2, 3]. Many heterotopic extraovarian sex-cord stromal tumors are granulosa cell tumors [2, 3]. To the best of our knowledge, only two cases of heterotopic extraovarian SLCTs, including our case, have been reported. Ovarian SLCTs mainly occur in young adult women, and less than 10% of patients with this malignancy are over 50 years of age [1]. In contrast, heterotopic extraovarian SLCTs, including our case, occur most frequently in elderly women, and no cases have been reported in young adults [3].

Ovarian SLCTs shows masculinization in approximately 40–60% of cases [1, 4, 5]. Ovarian SLCTs presenting with androgenic manifestation are more common among young adult women, whereas presentation with estrogenic manifestation is more frequent in elderly women [5]. Previous cases of heterotopic extraovarian SLCTs have not been described as having sex hormonal manifestations [3]. Similarly, our case was an elderly woman without sex hormonal manifestation. Thus, we suggest that heterotopic extraovarian SLCTs may present with no sex hormonal manifestations.

Recently, *DICER1* mutations have been reported in ovarian SLCTs [6–12]. *DICER1* hotspot-mutated ovarian SLCTs are characterized by androgenic effects, earlier age of onset, and frequent relapse compared with ovarian SLCTs without *DICER1* hotspot mutations [12, 13]. Previous studies of heterotopic extraovarian SLCTs did not report the examination of *DICER1* mutations [3]. Our case was an elderly woman who showed no androgenic manifestation and did not have *DICER1* hotspot mutations. Although our case exhibited heterotopic extraovarian SLCT, the clinical and molecular findings of our case were consistent with the characteristics of ovarian SLCTs without *DICER1* hotspot mutation.

Most of the mutations in *DICER1* are present in hotspots of exons 24 and 25 encoding the *DICER1* ribonuclease IIIb domain; however, a few exceptions have been reported [9]. Importantly, in our study, we did not examine the whole exon sequences of the *DICER1* gene. Accordingly, we could not exclude the potential occurrence of mutations in other exons. However, the characteristic androgenic effects and earlier age of onset of *DICER1* hotspot-mutated ovarian SLCTs was clarified by examination of hotspot mutations in a previous study [13]. Therefore, we believe that the features of heterotopic extraovarian SLCT may be consistent with the characteristics of ovarian SLCTs without *DICER1* hotspot mutations.

Ovarian SLCTs tend to recur earlier, within 1–2 years [1, 4]. In this case, the right ovarian tumor had been resected 18 years prior. If the right ovarian tumor from 18 years prior was SLCT, the patient may have shown earlier recurrence. Moreover, a sufficient number of tissue specimens were prepared from the right ovarian tumor, and these specimens contained only fibrothecoma components; no SLCT component was observed. Therefore, the mesenteric tumor was diagnosed as primary mesenteric SLCT, not late recurrence of the right ovarian tumor from 18 years prior.

In summary, we described the first case of heterotopic primary mesenteric SLCT without *DICER1* hotspot mutation. Our case was nonfunctional, similar to *DICER1* nonmutated ovarian SLCT.

## Abbreviations

CD56: Cluster of differentiation 56
SF-1: Steroidogenic factor-1
SLCT: Sertoli-Leydig cell tumor
WT-1: Wilms tumor 1

## References

[1] Young RH, Scully RE (1985). Ovarian Sertoli-Leydig cell tumors. A clinicopathological analysis of 207 cases. Am J Surg Pathol.

[2] Keitoku M, Konishi I, Nanbu K (1997). Extraovarian sex cord-stromal tumor: case report and review of the literature. Int J Gynecol Pathol.

[3] Trabelsi A, Ben Abdelkarim S, Hadfi M (2008). Primary mesenteric sertoli-leydig cell tumor: a case report and review of the literature. J Oncol.

[4] Zaloudek C, Norris HJ (1984). Sertoli-Leydig cell tumors of the ovary. A clinicopathological study of 64 intermediate and poorly differentiated neoplasms. Am J Surg Pathol.

[5] Gui T, Cao D, Shen K (2012). A clinicopathological analysis of 40 cases of ovarian Sertoli-Leydig cell tumors. Gynecol Oncol.

[6] Hill DA, Ivanovich J, Priest JR (2009). DICER1 mutations in familial pleuropulmonary blastoma. Science.

[7] Schultz KA, Pacheco MC, Yang J (2011). Ovarian sex cord-stromal tumors, pleuropulmonary blastoma and DICER1 mutations: a report from the International Pleuropulmonary Blastoma Registry. Gynecol Oncol.

[8] Rio Frio T, Bahubeshi A, Kanellopoulou C (2011). DICER1 mutations in familial multi-nodular goiter with and without ovarian Sertoli-Leydig cell tumors. JAMA.

[9] Heravi-Moussavi A, Anglesio MS, Cheng SW (2012). Recurrent somatic DICER1 mutations in nonepithelial ovarian cancers. N Engl J Med.

[10] Witkowski L, Mattina J, Schonberger S (2013). DICER1 hotspot mutations in non-epithelial gonadal tumours. Br J Cancer.

[11] Conlon N, Schultheis AM, Piscuoglio S (2015). A survey of DICER1 hotspot mutations in ovarian and testicular sex cord-stromal tumors. Mod Pathol.

[12] Goulvent T, Ray-Coquard I, Borel S (2016). DICER1 and FOXL2 mutations in ovarian sex cord-stromal tumours: a GINECO group study. Histopathology.

[13] Kato N, Kusumi T, Kamataki A (2017). DICER1 hotspot mutations in ovarian Sertoli-Leydig cell tumors: a potential association with androgenic effects. Hum Pathol.

